# Determining the Provincial and National Burden of Influenza-Associated Severe Acute Respiratory Illness in South Africa Using a Rapid Assessment Methodology

**DOI:** 10.1371/journal.pone.0132078

**Published:** 2015-07-08

**Authors:** Jillian Murray, Adam Cohen, Sibongile Walaza, Michelle Groome, Shabir Madhi, Ebrahim Variava, Kathleen Kahn, Halima Dawood, Stefano Tempia, Akhona Tshangela, Marietje Venter, Daniel Feikin, Cheryl Cohen

**Affiliations:** 1 Johns Hopkins International Vaccine Access Center, Baltimore, Maryland, United States of America; 2 Centers for Disease Control and Prevention, Pretoria, South Africa; 3 Centers for Disease Control and Prevention, Atlanta, United States of America; 4 National Institute of Communicable Diseases, Johannesburg, South Africa; 5 Medical Research Council: Respiratory and, Meningeal Pathogens Research Unit, Johannesburg, South Africa; 6 Schools of Public Health and Pathology, University of the Witwatersrand, Johannesburg, South Africa; 7 Klerksdorp Tshepong Hospital, Klerksdorp, South Africa; 8 University of KwaZulu-Natal, Durban, South Africa; 9 Umeå University, Umeå, Sweden; 10 INDEPTH Network, Accra, Ghana; 11 Pietermaritzburg Metropolitan Hospital Complex, Pietermaritzburg, South Africa; 12 Zoonoses Research Unit, Department of Medical Virology, University of Pretoria, Pretoria, South Africa; Georgia State University, UNITED STATES

## Abstract

Local disease burden data are necessary to set national influenza vaccination policy. In 2010 the population of South Africa was 50 million and the HIV prevalence was 11%. We used a previously developed methodology to determine severe influenza burden in South Africa.

Hospitalized severe acute respiratory illness (SARI) incidence was calculated, stratified by HIV status, for four age groups using data from population-based surveillance in one site situated in Gauteng Province for 2009–2011. These rates were adjusted for each of the remaining 8 provinces based on their prevalence of risk factors for pneumonia and healthcare-seeking behavior. We estimated non-hospitalized influenza-associated SARI from healthcare utilization surveys at two sites and used the percent of SARI cases positive for influenza from sentinel surveillance to derive the influenza-associated SARI rate. We applied rates of hospitalized and non-hospitalized influenza-associated SARI to census data to calculate the national number of cases. The percent of SARI cases that tested positive for influenza ranged from 7–17% depending on age group, year, province and HIV status. In 2010, there were an estimated 21,555 total severe influenza cases in HIV-uninfected individuals and 13,876 in HIV-infected individuals. In 2011, there were an estimated 29,892 total severe influenza cases in HIV-uninfected individuals and 17,289 in HIV-infected individuals. The incidence of influenza-associated SARI was highest in children <5 years and was higher in HIV-infected than HIV-uninfected persons in all age groups. Influenza virus was associated with a substantial amount of severe disease, especially in young children and HIV-infected populations in South Africa.

## Introduction

Influenza virus causes substantial disease in low- and middle-income countries each year, but data on the disease burden are limited [1]. Data on influenza from country-level surveillance can show the distribution of disease among the population and identify groups at high risk of infection. This information can subsequently help policy makers make evidence-based decisions on how to target influenza treatment and prevention programs, such as vaccination, and can lead to more effective allocation of resources within a country [2].

South Africa is a middle-income country, but there is great variation in socioeconomic status with some provinces that are more similar to low-income countries [3, 4]. This results in some populations within the country having a disproportionately higher level of exposure to risk factors for communicable disease. These include environmental risk factors, such as crowded living conditions and exposure to indoor air pollution, as well as biological risk factors, such as malnutrition and underlying infections [5–7]. These risk factors may drive the burden of influenza in South Africa to be greater than other countries with similar income level [3, 8, 9]. In particular, the high HIV prevalence in South Africa likely leads to higher numbers of severe influenza-associated illness and thus more hospitalized cases [9–15].

The influenza season in South Africa occurs in the austral winter months of May to August [14, 16, 17]. Influenza vaccine has been available in the public sector in South Africa for many years but coverage is only approximately 5%. The recommended target groups for annual vaccination include pregnant women, persons with underlying medical conditions (including HIV), children less than 5 years of age and persons over 65 years of age [18]. The estimation of national influenza burden is challenging particularly in low resource settings where data on the national number of consultations, hospitalizations and deaths are lacking in most instances. We estimated influenza-associated severe acute respiratory illness (SARI) by HIV status in South Africa using a rapid assessment methodology [2].

## Methods

The protocol for the SARI surveillance system was approved by the Research Ethics Committees of the Universities of the Witwatersrand and KwaZulu-Natal. The surveillance data used for the model was deemed non-research by the United States Centers for Disease Control and Prevention and did not need human subjects review by that institution. All data that was analyzed was de-identified and consent was obtained from all patients before they were enrolled into surveillance. Information from the surveys used was publically available.

### HIV-stratified model

**Fig 1:**
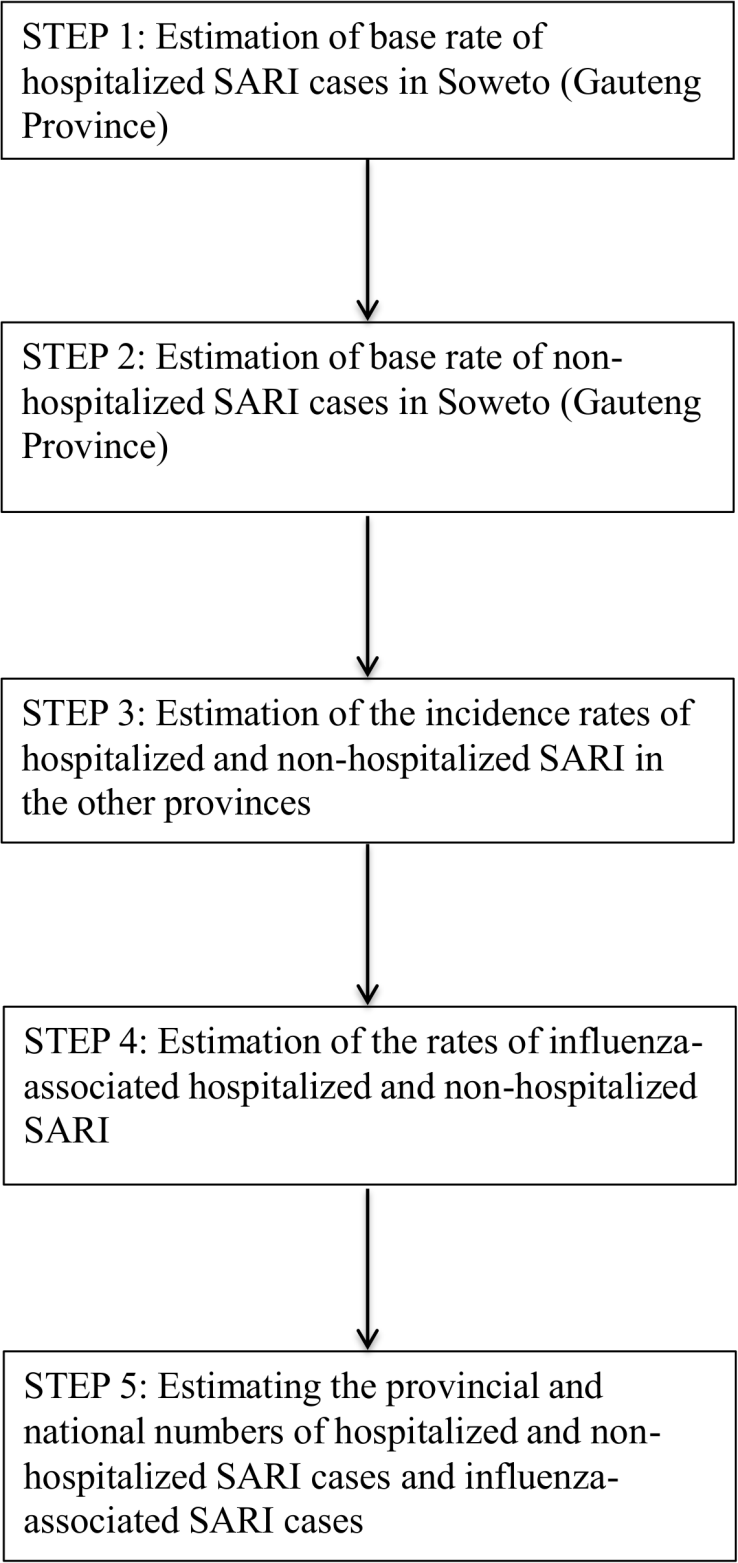
Flow diagram of methods. Visual depiction of the method used to calculate the rates and numbers of hospitalized and non-hospitalized SARI and influenza-associated SARI cases in of South Africa.

We utilized a multiplier model to estimate numbers of individuals with hospitalized and non-hospitalized influenza-associated SARI for each province in South Africa in four age groups (<5, 5–24, 25–44 and ≥45 years) for 2009–2011, stratified by HIV serostatus (Fig 1). Non-hospitalized SARI includes cases from the population that do not reach the hospital due to health care access barriers. This method was previously used in Kenya and Guatemala but was modified to include stratification by HIV serostatus to reflect the impact of HIV on influenza burden in South Africa [2].

The Centre for Respiratory Diseases and Meningitis at the National Institute of Communicable Diseases (NICD), Johannesburg, South Africa, began SARI surveillance in 2009 [19, 20]. Sentinel surveillance is conducted in four out of the nine South African provinces (Gauteng, KwaZulu-Natal, Mpumalanga and North West). Hospitalized children aged <5 years are enrolled in surveillance with physician-diagnosed lower respiratory tract infection, and individuals ≥5 years are enrolled if they meet a modified WHO case definition for SARI (sudden onset of fever with cough or sore throat and shortness of breath or difficulty breathing with 7 days from presentation to the hospital) [21]. Population-based surveillance is implemented at the Chris Hani Baragwanath Academic Hospital (CHBAH) in Gauteng Province. CHBAH is a large public academic hospital in the Soweto Township in Gauteng, South Africa. It serves approximately 1.4 million lower income South African individuals [12].

#### Estimation of the rate of hospitalized SARI cases in Soweto (Gauteng Province) (Fig 1, Step 1)

We estimated the total number of SARI hospitalizations using the number of enrolled SARI cases at CHBAH, adjusting for non-enrollment (refusal to participate and non-enrolment during weekends) by age groups and HIV status as previously described [12].

The rates of hospitalized SARI cases in Soweto were obtained by dividing the estimated number of SARI cases by the mid-year study population estimates by age group and HIV status. The HIV prevalence in the study population was obtained from the projections of the Actuarial Society of South Africa (ASSA) AIDS and Demographic model. The population of Soweto represents approximately 11% of the population of Gauteng Province (referred thereafter as the base Province). We assumed that the incidence rates for Soweto reflected the incidence rate for the province. At the time of this study, CHBAH was the only public hospital serving Soweto, where approximately 10% have private medical insurance ([22]. More than 80% of uninsured and 10% of insured persons seek care at public hospitals such as CHBAH. Therefore, the majority of individuals in Soweto that seek care do so at CHBAH [12].

#### Estimation of the rate of non-hospitalized SARI in Soweto (Gauteng Province) (Fig 1, Step 2)

We estimated the rate of non-hospitalized SARI in the base province using the data from two healthcare utilization surveys for SARI that were conducted in Soweto and Klerskdorp, North West Province in 2013. Non-hospitalized cases of SARI refer to cases of SARI identified in the population that had symptoms severe enough to be hospitalized but did not seek care at a hospital, for reasons such as limited access to healthcare. Briefly, between 4,000 and 6,000 community members were surveyed in each of the two sites as part of a cross-sectional door-to-door household survey and asked if they had a severe pneumonia in the past year (as defined as fever and cough and difficult breathing for 2–30 days or a physician diagnosis of pneumonia) and whether they were admitted to a hospital for the event. The ratio of those that sought care to those that did not was applied to the rate of hospitalized SARI to get the rate of non-hospitalized SARI in the base province [2].

#### Estimation of the rates of hospitalized and non-hospitalized SARI in the other provinces (Fig 1, Step 3)

Estimates of hospitalized SARI rates for the other eight provinces in South Africa were derived by adjusting the Soweto (Gauteng Province) SARI incidence rates for the provincial-level prevalence of known risk factors for pneumonia (from the South Africa Demographic and Health Survey and the ASSA model). Risk factors included exposure to indoor air pollution, crowding, malnutrition, low birth-weight and non-exclusive breastfeeding; the last three were only included as risk factors for children under five years of age [2, 8]. The relative risks of SARI associated with each risk factor were determined from the published literature [8]. In addition, we adjusted the provincial rates by the proportion of ARI seeking care in the given province to the proportion of ARI cases seeking care in the base province (Gauteng Province) using Health and Demographic Surveys (DHS) [4]. An upward adjustment factor (greater than one) resulted in a greater incidence of SARI in the province relative to Soweto (Gauteng Province). Similarly, a downward adjustment factor (less than one) resulted in a decrease in SARI incidence in the province relative to Soweto (Gauteng Province). The equations used for the Provincial adjustment are provided in the technical S1 Appendix.

The rates of non-hospitalized SARI in the remaining provinces were estimated using the provincial prevalence of known risk factors for pneumonia as described for the hospitalized cases. In addition, we adjusted the provincial rates by the proportion of ARI not seeking care in the given province to the proportion of ARI cases not seeking care in the base province (Gauteng Province) using Health and Demographic Surveys (DHS) [4].

#### Estimation of the rates of influenza-associated hospitalized and non-hospitalized SARI (Fig 1, Step 4)

The estimates of hospitalized and non-hospitalized SARI rates were subsequently multiplied by the percent of SARI associated with influenza virus from surveillance (i.e., the influenza detection rate) to give the incidence for influenza-associated SARI. Data on the percent of SARI due to influenza by age group in separate HIV-infected and-uninfected populations were pooled from all sentinel surveillance sites to give an average percentage that was used in the calculations for each province.

#### Estimating the provincial and national numbers of hospitalized and non-hospitalized SARI cases and influenza-associated SARI cases (Fig 1, Step 5)

The number of hospitalized and non-hospitalized SARI cases and influenza-associated SARI cases was obtained by multiplying the estimated rates by the mid-year population estimates. Provincial population estimates stratified by age group and HIV status were obtained from the ASSA 2008 population model.

### Non-HIV-stratified model

In an alternate method, we utilized a non-HIV-stratified model. To account for HIV prevalence in this model, we included HIV as a risk factor for SARI in calculating the provincial adjustment factors rather than estimating separate incidences for HIV-infected and-uninfected individuals. The relative risk for HIV was determined from the literature to be 7.3 for children under five years of age and 5.6 for children and adults over five years of age, and was used in previous applications of this methodology [2, 8, 23, 24]. The base SARI incidence rate in this method was also obtained from the population-based surveillance at CHBAH and the same healthcare utilization adjustments were made. Data on the proportion of SARI cases that were influenza-associated was pooled across surveillance sites to give an average percentage that was applied to all provinces.

### Estimation of the 95% confidence intervals

Confidence intervals were estimated by bootstrapping each parameter included in the calculations 1000 times, including the SARI rates in the base province, the influenza detection rate among SARI cases, the prevalence of the provincial risk factors, the proportion of SARI cases seeking care from the HUS in the base province and the proportion of ARI cases seeking care in each province form the DHS. The upper and lower limits of the 95% confidence intervals were the 2.5^th^ and 97.5^th^ percentile of these estimates, respectively.

## Results

### Base hospitalized SARI rates, Soweto, Gauteng

The highest rates of SARI in both HIV-infected and HIV-uninfected populations were among children less than five years of age, ranging from 2,253–5,507 per 100,000 in the HIV-infected population and 1,609–2,027 per 100,000 in the HIV-uninfected population over the three year study period [12]. The incidence rate of SARI in the HIV-infected population was consistently greater than the incidence in the HIV-uninfected population for all age groups. The rate ratio between HIV-infected and HIV-uninfected persons was highest in the 5–24 year old age group (range 14–31) and lowest in the under-5 year olds (range 1–3). SARI rates varied by year, with 2009 having the highest incidence rate for most age groups among both HIV-infected and HIV-uninfected populations.

### Base hospitalized influenza-associated SARI rates, Soweto, Gauteng

In 2009, when influenza A(H1N1)pdm09 was introduced into South Africa, the average percent of SARI from all surveillance sites that tested positive for influenza virus was 13% (152/1171) in HIV-uninfected individuals (all ages) compared to an average of 9% (91/1037) in HIV-infected individuals. In 2010, the average percent of SARI in which influenza virus was detected was 7% (114/1595) in HIV-uninfected individuals and 7% (116/1601) in HIV-infected individuals. In 2011, the average percent of SARI in which influenza virus was detected was 9% (193/2233) in HIV-uninfected individuals and 9% (127/1438) in HIV-infected individuals.

Similar to the base incidence of SARI, the base influenza-associated SARI incidence rate was greater among HIV-infected individuals for all three years and highest in children <5 year of age, ranging from 140–844 and 93–366 per 100,000 person-years in the HIV-infected and-uninfected populations, respectively, over the three year time period (Table 1). Among adults, influenza-associated SARI rates were highest among the oldest age group (>45 years) for HIV-infected (range 143–266 per 100,000 person-years) and HIV-uninfected populations (20–54 per 100,000 person-years) (Table 1).

**Table 1:** Estimated hospitalized influenza-associated severe acute respiratory illness (SARI) incidence (95% C.I.), stratified by HIV serostatus for each province and nationally, South Africa, 2009–2011. Data are rates per 100,000 persons.

Province	HIV-uninfected					HIV-infected				
	<5 years	5–24 years	25–44 years	≥45 years	All Ages	<5 years	5–24 years	25–44 years	≥45 years	All Ages
2009										
Eastern Cape	238(128–359)	11(<1–23)	8(1–26)	36(1–78)	38(15–73)	548(26–1381)	139(5–304)	100(3–224)	143(14–479)	154(6–328)
Free State	286(157–437)	13(<1–26)	8(1–30)	40(1–87)	46(17–84)	661(32–1655)	157(6–355)	112(4–253)	161(16–534)	186(7–368)
Gauteng (base)	252 (134–379)	12(<1–24)	8(1–27)	37(1–78)	40(14–73)	581(28–1445)	144(5–322)	103(3–236)	148(15–486)	164(6–325)
KwaZulu-Natal	354(194–531)	16(1–34)	11(2–37)	51(2–110)	57(23–106)	816(39–2041)	199(7–440)	143(5–332)	205(20–677)	230(9–473)
Limpopo	319(176–484)	14(<1–30)	9(1–34)	44(2–96)	51(23–98)	737(35–1846)	174(6–392)	125(4–277)	179(18–594)	207(8–419)
Mpumalanga	364(196–551)	17(1–35)	11(2–40)	54(2–117)	58(24–112)	840(40–2074)	211(8–470)	151(5–342)	216(22–719)	236(9–492)
Northern Cape	366(199–558)	15(<1–30)	10(1–34)	46(2–100)	59(22–104)	844(40–2118)	182(7–408)	130(4–293)	186(18–625)	237(9–444)
North West	287(155–436)	13(<1–27)	9(1–30)	41(1–88)	46(17–85)	662(31–1622)	160(6–356)	115(4–260)	164(16–546)	186(7–375)
Western Cape	326(175–494)	15(1–31)	10(1–34)	47(2–101)	52(18–93)	752(36–1868)	183(7–406)	131(5–299)	187(19–626)	211(6–406)
South Africa	302(164–458)	14(<1–29)	9(1–32)	42(2–93)	48(19–90)	715(34–1763)	177(6–386)	122(4–280)	266(17–580)	201(8–401)
2010										
Eastern Cape	93(20–188)	6(<1–14)	8(1–25)	20(1–47)	15(3–43)	236(19–701)	29(14–175)	105(34–190)	172(9–386)	105(25–234)
Free State	112(24–231)	7(<1–16)	9(1–28)	23(1–53)	18(3–50)	284(22–854)	32(16–196)	118(38–211)	194(10–422)	126(29–265)
Gauteng (base)	99(22–201)	6(<1–15)	8(1–25)	21(1–48)	16(3–44)	250(20–729)	30(15–178)	109(36–199)	178(9–389)	111(29–243)
KwaZulu-Natal	139(30–285)	8(<1–21)	11(1–35)	29(2–66)	22(4–63)	351(28–1034)	41(20–247)	151(49–272)	247(13–543)	156(37–334)
Limpopo	125(27–258)	7(<1–18)	10(1–30)	25(1–58)	20(4–57)	317(25–948)	36(18–217)	132(43–239)	216(11–479)	141(33–294)
Mpumalanga	143(31–290)	9(<1–22)	12(1–37)	31(2–70)	23(4–66)	361(29–1066)	43(22–266)	159(53–285)	261(13–577)	160(40–350)
Northern Cape	143(31–291)	8(<1–19)	10(1–32)	26(1–61)	23(1–61)	363(29–1069)	37(19–231)	137(45–240)	225(11–502)	161(34–316)
North West	113(25–228)	7(<1–17)	9(1–29)	23(1–54)	18(3–51)	285(22–831)	33(16–200)	121(39–218)	198(10–440)	126(3–51)
Western Cape	128(28–261)	8(<1–19)	10(1–32)	27(1–61)	21(3–56)	323(26–961)	38(19–228)	138(45–249)	226(11–498)	143(36–305)
South Africa	118(26–243)	7(<1–18)	9(1–30)	24(1–56)	19(3–53)	305(24–897)	36(18–217)	128(42–234)	209(11–464)	135(32–289)
2011										
Eastern Cape	127(49–229)	4(<1–10)	13(3–26)	31(10–56)	19(8–47)	140(18–478)	90(6–275)	126(48–213)	143(9–379)	91(32–260)
Free State	153(58–276)	4(<1–11)	14(4–30)	34(11–62)	22(10–55)	168(22–573)	101(7–310)	142(54–243)	160(10–425)	110(37–297)
Gauteng (base)	135(51–242)	4(<1–10)	13(3–27)	32(11–57)	20(9–49)	148(20–505)	93(6–286)	131(49–220)	147(9–388)	97(36–265)
KwaZulu-Natal	190(72–346)	5(<1–14)	18(5–37)	44(14–79)	28(12–69)	208(28–715)	129(9–395)	181(69–308)	204(12–549)	135(47–375)
Limpopo	171(64–306)	5(<1–12)	16(4–33)	38(13–70)	25(11–63)	188(25–648)	113(8–347)	158(59–274)	178(11–476)	122(41–329)
Mpumalanga	195(74–352)	6(<1–15)	19(5–40)	46(15–83)	29(13–73)	214(28–734)	137(9–418)	191(73–328)	216(13–573)	139(51–394)
Northern Cape	196(76–356)	5(<1–13)	16(4–35)	40(13–72)	29(12–68)	215(28–745)	118(8–359)	165(63–285)	186(11–495)	140(44–351)
North West	154(59–274)	4(<1–11)	14(4–30)	35(12–64)	23(10–56)	169(22–578)	104(7–326)	145(56–250)	164(10–436)	110(38–307)
Western Cape	175(66–309)	5(<1–13)	16(4–34)	40(13–73)	26(11–62)	192(25–658)	118(8–367)	166(62–284)	187(11–499)	125(47–338)
South Africa	162(61–293)	5(<1–12)	15(4–32)	37(12–67)	24(10–59)	179(24–618)	114(8–345)	153(59–265)	172(11–464)	117(41–322)

### Provincial hospitalized SARI and influenza-associated SARI rates

As the base province, Gauteng had a risk factor adjustment factor of 1. The Eastern Cape consistently had the largest downward adjustment factor (range 0.9–1.1), while Mpumalanga consistently had the largest upward adjustment factor (range 1.5–1.9; S1 Table). Across the three surveillance years studied, Mpumalanga and the Northern Cape consistently had the highest SARI incidence rate across both HIV-infected and uninfected groups and the four age groups studied (S2 Table). Rates for hospitalized influenza-associated SARI stratified by age and province are given in Table 1. The estimated incidence of influenza-associated SARI was consistently highest among the HIV-infected population across all age groups in all of the nine provinces for the surveillance years 2009–2011. Within both the HIV-infected and HIV-uninfected populations, the highest incidence of influenza-associated SARI was found in the <5 year olds for all provinces and surveillance years.

### National number of hospitalized and non-hospitalized cases of influenza-associated SARI

In 2009, the national number of hospitalized influenza-associated SARI cases was estimated to be 22,525 in HIV-uninfected individuals and 8,715 in HIV-infected individuals (Table 2). At two sites where healthcare utilization surveys were conducted, 61% of influenza-associated SARI cases were not hospitalized. In 2009, the total number of hospitalized and non-hospitalized influenza-associated SARI cases was estimated to be 47,711 in HIV-uninfected individuals and 17,993 in HIV-infected individuals (Table 2). In 2010 and 2011, there were fewer overall estimated influenza-associated SARI cases compared to 2009. In 2010, there were an estimated 21,555 total severe influenza cases in HIV-uninfected individuals and 13,876 in HIV-infected individuals (Table 2). In 2011, there were an estimated 29,892 total severe influenza cases in HIV-uninfected individuals and 17,289 in HIV-infected individuals (Table 2).

**Table 2:** Estimated national number (95% C.I.) of hospitalized and non-hospitalized influenza-associated severe acute respiratory illness (SARI) cases, stratified by HIV serostatus, South Africa, 2009–2011.

Age group (years)	HIV-infected			HIV-uninfected			HIV-infected and-uninfected-
	Number of hospitalized influenza cases	Number of non-hospitalized influenza cases	Total number of severe influenza cases	Number of hospitalized influenza cases	Number of non-hospitalized influenza cases	Total number of severe influenza cases	Total number of severe influenza cases
2009							
<5	1,510(72–3,768)	1,632(60–6,648)	3,142(132–10,416)	14,846(8044–22,414)	16,722(5,971–52,709)	31,568(14,015–75,123)	34,710(14,147–85,540)
5–24	1,783(65–3,956)	1,821(64–4,626)	3,604(129–8,582)	2,712(92–5,581)	2,890(112–6,860)	5,602(204–12,441)	9,206(333–21,023)
25–44	4,134(141–9,420)	4,421(185–11,603)	8,555(326–21,023)	1,005(145–3,524)	1,127(126–4,738)	2,132(271–8,262)	10,687(597–29,285)
≥45	1,288(129–4,275)	1,404(108–5,058)	2,692(237–9,333)	3,962(141–8,562)	4,447(159–10,981)	8,409(300–19,543)	11,101(537–28,877)
All Ages	8,715(406–21,419)	9,278(417–27,935)	17,993(823–49,354)	22,525(8,423–40,082)	25,186(6,368–75,287)	47,711(14,791–115,369)	65,704(15,614–164,723)
2010							
<5	615(49–1,813)	664(31–3,319)	1,279(80–5,132)	5,809(1,267–11,861)	6,536(965–23,707)	12,345(2,232–35,568)	13,624(2,312–40,699)
5–24	364(182–2,205)	373(142–2,825)	737(324–5,030)	1,381(63–3,446)	1,471(61–3,968)	2,852(124–7,414)	3,589(447–12,444)
25–44	4,442(1,452–8,015)	4,743(1,302–9,750)	9,185(2,754–17,765)	1,038(101–3,328)	1,161(90–3,969)	2,199(191–7,297)	11,384(2,945–25,062)
≥45	1,279(84–3,660)	1,396(76–4,361)	2,675(160–8,021)	1,958(122–5,283)	2,201(113–6,945)	4,159(235–12,128)	6,834(395–20,148)
All Ages	6,700(1,767–15,693)	7,176(1,551–20,254)	13,876(3,318–35,947)	10,186(1,553–23,919)	11,369(1,228–38,488)	21,555(2,781–62,407)	35,431(6,098–98,353)
2011							
<5	344(45–1,177)	372(27–1,921)	716(72–3,098)	7,928(2,994–14,256)	8,906(2,475–28,131)	16,834(5,469–42,387)	17,550(5,541–45,486)
5–24	1,133(76–3,479)	1,155(67–4,003)	2,288(143–7,482)	898(51–2,364)	960(46–2,835)	1,858(97–5,199)	4,146(240–12,682)
25–44	5,436(2,064–9,259)	5,792(2,007–11,699)	11,228(4,071–20,958)	1,735(458–3,644)	1,936(356–4,652)	3,671(814–8,296)	14,899(4,885–29,254)
≥45	1,462(89–3,895)	1,595(80–4,872)	3,057(169–8,767)	3,545(1,165–6,444)	3,984(1,294–8,454)	7,529(2,459–14,898)	10,586(2,628–23,665)
All Ages	8,375(2,274–17,811)	8,914(2,181–22,494)	17,289(4,455–40,305)	14,106(4,668–26,708)	15,786(4,171–44,072)	29,892(8,839–70,780)	47,181(13,294–111,085)

### Non-HIV-stratified model

In our alternate method in which HIV was adjusted for as a risk factor rather than a stratifying variable, the greatest impact on the adjustment factors was for the 25–44 year old age group where the HIV prevalence is highest. The Eastern Cape Province still consistently had the largest downward adjustment factor, except in the 25–44 year old age group where Limpopo had the largest downward adjustment. However, KwaZulu-Natal Province consistently had the largest upward adjustment factor across all age groups, due to the high HIV prevalence in that province. The results from method 2 yielded similar incidence of influenza-associated SARI and number of hospitalized cases across the age groups and years included in the analysis (S3 Table). The non-stratified alternate method estimated a total of 74,558 cases of hospitalized influenza-associated SARI for 2009–2011, only 6% more than the 70,607 cases estimated by the stratified model for the same years.

## Discussion

Influenza-associated SARI represents a substantial burden of disease in South Africa. Using a rapid assessment methodology, we found that the highest rate was seen in children <5 years of age irrespective of HIV status. Among adults, the highest number was seen in HIV-infected adults aged 25–44 years. Previous studies of influenza in South Africa have not calculated a national burden [25–28] and have not estimated the non-hospitalized SARI burden. Our methodology allowed estimation of the number of cases in each province by age group and HIV serostatus, which can support decisions on targeted interventions within the confines of limited resources.

Our study highlights the important role that HIV infection plays when estimating severe influenza burden in settings with high HIV prevalence. Among the age group with the highest HIV prevalence (25–44 year olds), HIV-infected persons accounted for 80% of the national burden of influenza-associated SARI, despite representing only 30% of the national population [29]. HIV-specific incidences for influenza-associated disease in South Africa have been reported previously to be high in HIV-infected individuals [11, 12]. A study in children 2–60 months of age in 1997 reported influenza-associated severe lower respiratory tract infection incidence rates of 1,268 and 148 per 100,000 for HIV-infected and HIV-uninfected children, respectively [11]. More recently, an analysis of population-based data from CHBAH found influenza-associated lower respiratory tract infections (LRTI) to be highest in the HIV-infected population [12].

Some countries may not be able to calculate HIV-specific influenza incidence rates due to lack of reliable HIV-stratified influenza data in the population. In the non-HIV-stratified alternate method, we derived similar estimates of burden using a model incorporating adjustment for HIV prevalence by province. This suggests that HIV-specific influenza incidence rates are not necessary to derive valid overall burden estimates because HIV prevalence by province is available in most African countries through Demographic and Health Surveys or Multicluster Indicator Surveys [4].

There were several limitations to our methodology. Our study relies on extrapolating incidence rates from one surveillance site in one province to the rest of the provinces of South Africa. Although a high percentage of the base province is under surveillance from this one large surveillance site and we adjusted this incidence based on province-level risk factor for SARI, there are likely additional unmeasured factors that can lead to variation in rates regionally. We assumed that the relative risk of SARI for the different risk factors determined for the populations in the literature are the same relative risks for the population of South Africa, which might not be the case [8, 23, 24]. Second, the denominator estimates also might have been inaccurate. We used the ASSA 2008 population model to estimate the population and HIV prevalence by province and age group. These population estimates are extrapolations from the 2001 census. Third, the healthcare utilization surveys used to calculate non-hospitalized cases was undertaken in only two sites in two provinces. Moreover, the surveys did not stratify health-seeking by HIV status or age, which is likely to influence health-seeking behavior. Fourth, these estimates reflect the dsease burden in the public sector; however, a similar study using a different modelling approach in the private health sector of South Africa found similar incidence rates of influenza-associated respiratory hospitalizations [30]. Lastly, we assume that the percent of influenza-associated SARI is the same across all age groups, provinces and HIV status.

## Conclusion

This rapid assessment methodology has been used in Kenya, Guatemala and now South Africa [2]. Other countries that have influenza surveillance in place can also implement this methodology to estimate their national burden of severe influenza disease. We found that provinces in South Africa vary in the prevalence of important risk factors for SARI and influenza and that these impact the burden of severe disease. Current recommendations in South Africa include the vaccination of HIV-infected individuals with the seasonal influenza vaccine; however, the vaccine tends to be under-utilized in this population and in South Africa in general[31]. The results of this rapid assessment can be used to focus efforts targeting influenza vaccination by demonstrating the distribution of disease across South Africa.

## Supporting Information

S1 Table Provincial adjustment factors for severe acute respiratory illness (SARI) healthcare-seeking behavior, 2009–2011. (DOCX)

S2 Table Estimated hospitalized severe acute respiratory illness (SARI) incidence (95% C.I.) stratified by HIV serostatus for South Africa, 2009–2011. Data are rates per 100,000 persons.
(DOCX)

S3 Table Influenza-associated hospitalized severe acute respiratory illness (SARI) incidence and number of cases in each province for 2009–2011, stratified by HIV serostatus. (DOCX)

S1 Appendix Equations used in calculation of annual number of cases of influenza-associated severe acute respiratory illness (SARI). (DOCX)
