# Supplementary material for: Determining the Provincial and National Burden of Influenza-Associated Severe Acute Respiratory Illness in South Africa Using a Rapid Assessment Methodology
Source: PLoS One. 2015 Jul 8;10(7):e0132078. doi: 10.1371/journal.pone.0132078 (PMC4496064; doi:10.1371/journal.pone.0132078)
Supplement: S1 Table — (DOCX) [file pone.0132078.s001.docx]

**S1 Table.** Provincial adjustment factors for severe acute respiratory illness (SARI) healthcare-seeking behavior, 2009-2011

| **Province** | **Adjustment for provincial-level risk factors for SARI (with HIV)** | **Adjustment for provincial-level risk factors for SARI (without HIV)** | **Adjustment for provincial-level risk factors for SARI (with HIV)** | **Adjustment for provincial-level risk factors for SARI (without HIV)** |
| --- | --- | --- | --- | --- |
|  | **Children <5 years** | | **Children and adults 5-24 years** | |
| **Eastern Cape** | 0.92 | 0.94 | 1.00 | 0.97 |
| **Free State** | 1.16 | 1.14 | 1.15 | 1.09 |
| **Gauteng (base)** | 1.00 | 1.00 | 1.00 | 1.00 |
| **KwaZulu-Natal** | 1.54 | 1.40 | 1.56 | 1.39 |
| **Limpopo** | 1.14 | 1.27 | 1.15 | 1.21 |
| **Mpumalanga** | 1.51 | 1.45 | 1.56 | 1.47 |
| **Northern Cape** | 1.34 | 1.45 | 1.16 | 1.26 |
| **North West** | 1.16 | 1.14 | 1.17 | 1.11 |
| **Western Cape** | 1.10 | 1.29 | 1.11 | 1.27 |
|  | **Adults 25-44 years** | | **Adults ≥45 years** | |
| **Eastern Cape** | 1.10 | 0.97 | 0.96 | 0.97 |
| **Free State** | 1.28 | 1.09 | 1.16 | 1.09 |
| **Gauteng (base)** | 1.00 | 1.00 | 1.00 | 1.00 |
| **KwaZulu-Natal** | 2.09 | 1.39 | 1.61 | 1.39 |
| **Limpopo** | 0.92 | 1.21 | 1.02 | 1.21 |
| **Mpumalanga** | 1.89 | 1.47 | 1.55 | 1.47 |
| **Northern Cape** | 0.85 | 1.26 | 1.12 | 1.26 |
| **North West** | 1.32 | 1.11 | 1.23 | 1.11 |
| **Western Cape** | 0.64 | 1.27 | 1.01 | 1.27 |
