# Supplementary material for: Determining the Provincial and National Burden of Influenza-Associated Severe Acute Respiratory Illness in South Africa Using a Rapid Assessment Methodology
Source: PLoS One. 2015 Jul 8;10(7):e0132078. doi: 10.1371/journal.pone.0132078 (PMC4496064; doi:10.1371/journal.pone.0132078)
Supplement: S2 Table — Data are rates per 100,000 persons. (DOCX) [file pone.0132078.s002.docx]

**S2 Table.** Estimated hospitalized severe acute respiratory illness (SARI) incidence (95% C.I.) stratified by HIV serostatus for South Africa, 2009-2011. Data are rates per 100,000 persons.

| **Province** | **HIV-uninfected** | | | | | **HIV-infected** | | | | |
| --- | --- | --- | --- | --- | --- | --- | --- | --- | --- | --- |
|  | **<5 years** | **5-24 years** | **25-44 years** | **≥45 years** | **All Ages** | **<5 years** | **5-24 years** | **25-44 years** | **≥45 years** | **All Ages** |
| **2009** | | | | | | | | | | |
| **Eastern Cape** | 1909 (1720-2095) | 67 (59-75) | 73 (63-83) | 249 (224-279) | 316 (284-349) | 5189 (4585-5873) | 914 (807-1029) | 1363 (1253-1483) | 1979 (1799-2182) | 1501 (1360-1655) |
| **Free State** | 2304 (2113-2504) | 75 (66-84) | 82 (71-93) | 279 (253-309) | 359 (326-393) | 6263 (5544-7052) | 1027 (914-1154) | 1532 (1422-1650) | 2224 (2021-2448) | 1717 (1569-1877) |
| **Gauteng (base)** | 2026 (1956-2102) | 69 (62-75) | 75 (67-84) | 257 (240-275) | 312 (296-329) | 5507 (5031-5983) | 944 (862-1028) | 1408 (1360-1458) | 2044 (1918-2165) | 1581 (1502-1660) |
| **KwaZulu-Natal** | 2845 (1645-3070) | 95 (85-106) | 104 (91-118) | 356 (325-389) | 462 (426-502) | 7732 (6936-8611) | 1307 (1177-1458) | 1951 (1819-2090) | 2831 (2608-3098) | 2170 (1998-2360) |
| **Limpopo** | 2569 (2356-2799) | 83 (74-93) | 91 (79-103) | 311 (282-342) | 430 (392-471) | 6982 (6221-7920) | 1143 (1017-1270) | 1705 (1583-1832) | 2475 (2254-2717) | 1922 (1756-2103) |
| **Mpumalanga** | 2929 (2715-3162) | 101 (90-112) | 110 (96-125) | 377 (345-413) | 490 (450-533) | 7963 (7116-8932) | 1384 (1240-1534) | 2064 (1932-2224) | 2996 (2748-3274) | 2299 (2118-2508) |
| **Northern Cape** | 2943 (2710-3195) | 87 (78-98) | 95 (83-108) | 324 (296-356) | 450 (411-492) | 7998 (7135-8906) | 1192 (1057-1335) | 1779 (1642-1916) | 2582 (2352-2828) | 2081 (1898-2269) |
| **North West** | 2310 (2127-2519) | 77 (68-86) | 83 (72-95) | 286 (258-317) | 364 (332-401) | 6279 (5566-7046) | 1050 (944-1170) | 1567 (1448-1695) | 2275 (2066-2501) | 1767 (1613-1933) |
| **Western Cape** | 2619 (2418-2838) | 87 (78-98) | 95 (84-108) | 326 (295-358) | 390 (356-426) | 7119 (6343-7959) | 1198 (1066-1338) | 1787 (1652-1923) | 2593 (2357-2827) | 1995 (1826-2167) |
| **South Africa** | 2438 (2263-2628) | 82 (73-91) | 88 (77-99) | 301 (275-330) | 388 (357-422) | 6701 (6001-7476) | 1140 (1022-1268) | 1675 (1569-1789) | 2419 (2225-2634) | 1876 (1732-2033) |
| **2010** | | | | | | | | | | |
| **Eastern Cape** | 1517 (1376-1672) | 36 (31-41) | 81 (71-92) | 215 (192-239) | 250 (224-277) | 3078 (2682-3527) | 804 (704-909) | 1296 (1185-1417) | 2119 (1918-2347) | 1367 (1234-1512) |
| **Free State** | 1830 (1668-2012) | 40 (34-46) | 91 (80-103) | 241 (219-268) | 287 (259-317) | 3715 (3205-4273) | 904 (793-1008) | 1457 (1331-1572) | 2382 (2169-2610) | 1584 (1434-1731) |
| **Gauteng (base)** | 1609 (1551-1670) | 37 (32-41) | 84 (74-93) | 222 (207-239) | 251 (237-265) | 3267 (2900-3627) | 830 (749-909) | 1338 (1287-1383) | 2189 (2063-2312) | 1467 (1391-1539) |
| **KwaZulu-Natal** | 2260 (2096-2442) | 51 (44-58) | 116 (102-131) | 307 (282-339) | 365 (336-399) | 4587 (3995-5200) | 1151 (1021-1272) | 1854 (1726-1988) | 3032 (2793-3293) | 1975 (1813-2142) |
| **Limpopo** | 2041 (1878-2225) | 45 (38-52) | 101 (89-115) | 269 (243-296) | 340 (309-373) | 4142 (3580-4756) | 1006 (895-1115) | 1621 (1498-1738) | 2650 (2424-2896) | 1711 (1559-1864) |
| **Mpumalanga** | 2327 (2153-2527) | 54 (47-62) | 123 (108-139) | 325 (295-358) | 387 (354-424) | 4724 (4134-5315) | 1218 (1083-1359) | 1962 (1830-2108) | 3209 (2937-3485) | 2081 (1912-2261) |
| **Northern Cape** | 2338 (2155-2548) | 47 (40-54) | 106 (93-119) | 280 (254-308) | 358 (326-393) | 4745 (4112-5420) | 1049 (933-1176) | 1691 (1558-1815) | 2765 (2521-3020) | 1892 (1723-2059) |
| **North West** | 1835 (1678-2013) | 41 (35-47) | 93 (81-106) | 247 (224-274) | 293 (266-324) | 3725 (3222-4269) | 924 (811-1033) | 1490 (1366-1621) | 2436 (2208-2687) | 1630 (1477-1793) |
| **Western Cape** | 2081 (1912-2257) | 47 (40-54) | 106 (93-119) | 282 (256-313) | 315 (286-346) | 4223 (3666-4780) | 1054 (931-1175) | 1698 (1578-1827) | 2777 (2536-3029) | 1862 (1710-2021) |
| **South Africa** | 1938 (1797-2094) | 44 (38-50) | 98 (86-110) | 260 (237-286) | 309 (283-337) | 3972 (3466-4504) | 1003 (890-1114) | 1593 (1486-1702) | 2590 (2384-2810) | 1716 (1578-1857) |
| **2011** | | | | | | | | | | |
| **Eastern Cape** | 1848 (1674-2026) | 30 (26-35) | 79 (70-90) | 222 (199-246) | 285 (256-314) | 2123 (1772-2474) | 941 (803-1090) | 1296 (1183-1410) | 2008 (1806-2231) | 1350 (1210-1496) |
| **Free State** | 2231 (2025-2430) | 34 (29-39) | 89 (79-101) | 249 (225-275) | 327 (295-359) | 2562 (2164-3007) | 1057 (905-1234) | 1456 (1333-1579) | 2257 (2040-2496) | 1558 (1404-1720) |
| **Gauteng (base)** | 1961 (1893-2029) | 31 (27-35) | 82 (74-90) | 229 (213-246) | 281 (267-296) | 2253 (1938-2567) | 971 (841-1114) | 1338 (1287-1397) | 2074 (1935-2219) | 1441 (1357-1532) |
| **KwaZulu-Natal** | 2754 (2543-2957) | 43 (37-49) | 114 (101-127) | 318 (288-346) | 419 (384-453) | 3164 (2664-3676) | 1346 (1154-1557) | 1854 (1725-1997) | 2873 (2627-3141) | 1943 (1771-2132) |
| **Limpopo** | 2487 (2290-2723) | 38 (32-43) | 99 (88-111) | 278 (253-303) | 391 (357-429) | 2857 (2408-3316) | 1176 (1008-1375) | 1621 (1502-1749) | 2511 (2269-2760) | 1678 (1520-1850) |
| **Mpumalanga** | 2836 (2623-3069) | 45 (39-52) | 120 (107-134) | 336 (306-366) | 442 (406-481) | 3258 (2767-3765) | 1424 (1220-1648) | 1962 (1830-2124) | 3040 (2775-3320) | 2048 (1870-2250) |
| **Northern Cape** | 2849 (2609-3099) | 39 (34-45) | 104 (91-117) | 290 (262-320) | 406 (369-445) | 3272 (2751-3829) | 1227 (1059-1427) | 1690 (1567-1823) | 2619 (2380-2880) | 1844 (1678-2025) |
| **North West** | 2236 (2047-2444) | 35 (29-40) | 91 (80-103) | 255 (230-281) | 337 (306-370) | 2569 (2148-2998) | 1081 (915-1257) | 1489 (1380-1619) | 2308 (2080-2541) | 1598 (1448-1764) |
| **Western Cape** | 2536 (2329-2754) | 39 (34-45) | 104 (93-116) | 291 (263-319) | 355 (323-388) | 2913 (2449-3400) | 1232 (1054-1416) | 1698 (1570-1845) | 2631 (2391-2891) | 1833 (1672-2012) |
| **South Africa** | 2363 (2187-2546) | 37 (32-42) | 96 (86-107) | 268 (244-293) | 352 (323-382) | 2741 (2315-3177) | 1173 (1006-1359) | 1594 (1488-1712) | 2453 (2241-2680) | 1687 (1543-1845) |
