# Supplementary material for: Determining the Provincial and National Burden of Influenza-Associated Severe Acute Respiratory Illness in South Africa Using a Rapid Assessment Methodology
Source: PLoS One. 2015 Jul 8;10(7):e0132078. doi: 10.1371/journal.pone.0132078 (PMC4496064; doi:10.1371/journal.pone.0132078)
Supplement: S3 Table — (DOCX) [file pone.0132078.s003.docx]

**S3 Table.** Influenza-associated hospitalized severe acute respiratory illness (SARI) incidence and number of cases in each province for 2009-2011, stratified by HIV serostatus.

| **Province** | **Influenza-associated hospitalized SARI incidence rate (per 100,000 persons)** | | | | **Number of influenza-associated SARI hospitalized cases** | | | |
| --- | --- | --- | --- | --- | --- | --- | --- | --- |
|  | **<5 years** | **5-24 years** | **25-44 years** | **≥45 years** | **<5 years** | **5-24 years** | **25-44 years** | **≥45 years** |
| **2009** |  |  |  |  |  |  |  |  |
| **Eastern Cape** | 229  (137-380) | 21  (7-38) | 47  (1-95) | 42  (2-105) | 1652  (985-2742) | 647  (225-1192) | 733  (21-1486) | 540  (10-1334) |
| **Free State** | 287  (168-455) | 24  (8-44) | 54  (2-109) | 51  (2-126) | 808  (473-1281) | 272  (94-505) | 453  (13-910) | 327  (12-809) |
| **Gauteng (base)** | 248  (153-393) | 21  (7-38) | 42  (1-83) | 44  (2-108) | 2462  (1515-3902) | 716  (247-1328) | 1606  (45-3155) | 1050  (37-2565) |
| **KwaZulu-Natal** | 381  (232-614) | 32  (11-60) | 88  (2-175) | 71  (3-175) | 4124  (2509-6646) | 1482  (517-2762) | 2505  (70-4965) | 1334  (48-3290) |
| **Limpopo** | 284  (176-469) | 24  (8-44) | 39  (1-80) | 45  (2-111) | 1923  (1192-3175) | 647  (224-1199) | 532  (15-1085) | 412  (15-1020) |
| **Mpumalanga** | 374  (231-603) | 32  (11-60) | 80  (2-164) | 68  (2-168) | 1548  (955-2496) | 535  (184-993) | 832  (23-1700) | 448  (16-1099) |
| **Northern Cape** | 331  (199-543) | 24  (8-45) | 36  (1-73) | 49  (2-122) | 384  (231-630) | 106  (37-198) | 113  (3-230) | 122  (5-301) |
| **North West** | 288  (175-478) | 24  (8-46) | 56  (2-115) | 54  (2-134) | 975  (591-1618) | 326  (111-617) | 566  (16-1161) | 388  (14-962) |
| **Western Cape** | 274  (159-440) | 23  (8-43) | 27  (1-55) | 44  (2-110) | 1329  (772-2132) | 416  (142-784) | 460  (13-934) | 552  (20-1361) |
| **South Africa** | 297  (181-482) | 25  (9-47) | 54  (2-108) | 52  (2-128) | 15206  (9224-24,622) | 5147  (1781-9578) | 7799  (219-15,626) | 5173  (187-12,741) |
| **2010** |  |  |  |  |  |  |  |  |
| **Eastern Cape** | 99  (33-190) | 8  (<1-22) | 49  (20-87) | 36  (1-76) | 708  (240-1363) | 250  (15-676) | 793  (326-1420) | 457  (15-979) |
| **Free State** | 121  (41-235) | 9  (1-25) | 56  (23-97) | 43  (1-92) | 338  (116-657) | 105  (6-279) | 470  (192-817) | 277  (9-602) |
| **Gauteng (base)** | 104  (38-199) | 8  (<1-22) | 44  (18-77) | 37  (1-81) | 1013  (369-1944) | 283  (17-760) | 1654  (696-2910) | 908  (29-1991) |
| **KwaZulu-Natal** | 159  (57-309) | 13  (1-34) | 91  (37-161) | 60  (2-133) | 1712  (614-3325) | 577  (35-1561) | 2639  (1074-4647) | 1139  (36-2537) |
| **Limpopo** | 121  (43-239) | 9  (1-25) | 41  (17-72) | 38  (1-81) | 819  (293-1626) | 251  (15-680) | 583  (238-1032) | 353  (11-758) |
| **Mpumalanga** | 158  (56-301) | 13  (1-34) | 83  (34-149) | 57  (2-126) | 651  (230-1237) | 209  (13-569) | 881  (362-1574) | 384  (12-847) |
| **Northern Cape** | 139  (49-269) | 9  (1-25) | 38  (15-66) | 41  (1-90) | 160  (56-309) | 42  (3-113) | 120  (49-213) | 104  (3-227) |
| **North West** | 122  (42-236) | 9  (1-26) | 58  (23-103) | 45  (1-98) | 417  (144-806) | 127  (8-347) | 588  (234-1049) | 331  (11-723) |
| **Western Cape** | 117  (40-226) | 9  (1-24) | 28  (12-51) | 37  (1-82) | 565  (191-1089) | 163  (10-445) | 486  (198-867) | 475  (15-1049) |
| **South Africa** | 125  (44-243) | 10  (1-27) | 56  (23-99) | 43  (1-95) | 6383  (2252-12,356) | 2006  (122-5431) | 8215  (3369-14,528) | 4428  (141-9712) |
| **2011** |  |  |  |  |  |  |  |  |
| **Eastern Cape** | 130  (46-215) | 5  (<1-14) | 38  (22-76) | 31  (18-77) | 932  (328-1540) | 155  (7-437) | 646  (381-1290) | 404  (237-997) |
| **Free State** | 159  (55-259) | 6  (<1-16) | 44  (25-88) | 37  (22-92) | 445  (155-724) | 66  (3-186) | 372  (216-754) | 246  (146-609) |
| **Gauteng (base)** | 138  (47-224) | 5  (<1-14) | 34  (20-68) | 32  (20-80) | 1328  (457-2153) | 180  (8-515) | 1286  (759-2564) | 821  (498-2041) |
| **KwaZulu-Natal** | 210  (73-348) | 8  (<1-23) | 71  (41-141) | 52  (32-130) | 2260  (790-3750) | 361  (15-1035) | 2111  (1224-4172) | 1016  (610-2525) |
| **Limpopo** | 161  (57-276) | 6  (<1-17) | 32  (19-64) | 33  (20-80) | 1095  (392-1885) | 157  (7-454) | 478  (285-962) | 316  (191-769) |
| **Mpumalanga** | 208  (74-348) | 8  (<1-23) | 65  (39-129) | 50  (30-122) | 854  (306-1429) | 131  (6-384) | 705  (420-1400) | 345  (206-842) |
| **Northern Cape** | 186  (65-303) | 6  (<1-17) | 29  (17-59) | 36  (22-88) | 212  (74-345) | 26  (1-75) | 96  (57-192) | 93  (56-227) |
| **North West** | 161  (56-266) | 6  (<1-17) | 45  (27-91) | 39  (23-97) | 554  (194-915) | 80  (3-232) | 465  (276-939) | 296  (177-728) |
| **Western Cape** | 158  (54-254) | 6  (<1-16) | 22  (13-45) | 32  (19-80) | 760  (262-1221) | 103  (5-292) | 382  (224-774) | 427  (256-1056) |
| **South Africa** | 166  (58-276) | 6  (<1-18) | 44  (26-87) | 38  (23-94) | 8439  (2956-13,962) | 1258  (56-3609) | 6540  (3841-13,048) | 3964  (2376-9795) |
