# Supplementary material for: Determining the Provincial and National Burden of Influenza-Associated Severe Acute Respiratory Illness in South Africa Using a Rapid Assessment Methodology
Source: PLoS One. 2015 Jul 8;10(7):e0132078. doi: 10.1371/journal.pone.0132078 (PMC4496064; doi:10.1371/journal.pone.0132078)
Supplement: S1 Appendix — (DOCX) [file pone.0132078.s004.docx]

**S1 Appendix**. Equations used in calculation of annual number of cases of influenza-associated severe acute respiratory illness (SARI).

**Equation 1: Rate of hospitalized and non-hospitalized SARI in base province (Gauteng Province)**

$${RS}_{H,B}=\frac{{SARI}_{H,B}}{{Pop}_{B}}$$

Where:

${RS}_{H,B}$ = Base rate of hospitalized SARI

${SARI}_{H,B}$ = Number of total cases meeting SARI case definition hospitalized at Chris Hani Baragwanath Academic Hospital (CHBAH) (Gauteng Province, Soweto)

${Pop}_{B}$ = Population of surveillance catchment area (Soweto)

$${RS}_{NH,B}=\left( \frac{{RS}_{H,B}}{{HUS}_{B}} \right)-{RS}_{H,B}$$

Where:

${RS}_{NH,B}$ = Base rate of non-hospitalized SARI
${HUS}_{B}$ = Proportion of all SARI cases that are hospitalized in the base province from Healthcare Utilization Survey (HUS)

**Equation 2a: Calculation of adjustment for risk factors at the provincial level for base rate hospitalized and non-hospitalized SARI**

$${Adj}_{Y}=\left( 1+\sum_{i} \left( P_{i,Y}-P_{i,B} \right)\times\left( {RR}_{i}-1 \right) \right)$$

Where:

${Adj}_{Y}$ = Adjustment factor for province Y for risk factors of SARI

$P_{i,Y}$ = Prevalence of risk factor *i* in province Y

$P_{i,B}$= Prevalence of risk factor *i* in base province

${RR}_{i}$ = Relative risk of SARI due to risk factor *i*

**Equation 2b: Rate of hospitalized and non-hospitalized SARI in province after adjustment for risk factors and healthcare-seeking behavior**

$${RS}_{H,Y}={RS}_{H,B} \times{Adj}_{Y} \times\frac{{DHS}_{H,Y}}{{DHS}_{H,B}}$$

Where:

${RS}_{H,Y}$ = Rate of hospitalized SARI in province Y

${DHS}_{H,Y}$ = Proportion of ARI cases seeking care in province Y (from DHS)

${DHS}_{H,B}$ = Proportion of ARI cases seeking care in base province (from DHS)

$${RS}_{NH,Y}={RS}_{NH,B} \times{Adj}_{Y} \times\frac{{DHS}_{NH,Y}}{{DHS}_{NH,B}}$$

Where:

${RS}_{NH,Y}$ = Rate of non-hospitalized SARI in province Y

${DHS}_{NH,Y}$ = Proportion of ARI cases not seeking care in province Y (from DHS)

${DHS}_{NH,B}$ = Proportion of ARI cases not seeking care in base province (from DHS)

**Equation 3: Number of hospitalized and non-hospitalized SARI cases in province**

$${NS}_{H,Y}={RS}_{H,Y} \times P{op}_{Y}$$

$${NS}_{NH,Y}={RS}_{NH,Y} \times P{op}_{Y}$$

Where:

${NS}_{H,Y}$= Number of hospitalized SARI cases in province Y (including base province)

${NS}_{NH,Y}$= Number of non-hospitalized SARI cases in province Y (including base province)

$P{op}_{Y}$= Population in province Y (including base province)

**Equation 4: Rate of hospitalized and non-hospitalized influenza-associated SARI in province**

$${RF}_{H,Y}={RS}_{H,Y} \times F$$

$${RF}_{NH,Y}={RS}_{NH,Y} \times F$$

Where:

${RF}_{H,Y}$= Rate of hospitalized influenza-associated SARI in province Y (including base

province)

${RF}_{NH,Y}$= Rate of non-hospitalized influenza-associated SARI in province Y (including base

province)

$F$ = Proportion of hospitalized SARI cases testing positive for influenza (from all SARI

surveillance sites)

**Equation 5: Number of hospitalized and non-hospitalized influenza-associated SARI cases in province**

$${NF}_{H,Y}={RF}_{H,Y} \times P{op}_{Y}$$

$${NF}_{NH,Y}={RF}_{NH,Y} \times P{op}_{Y}$$

Where:

${NF}_{H,Y}$= Number of hospitalized influenza-associated SARI cases in province Y (including

base province)

${NF}_{NH,Y}$= Number of non-hospitalized influenza-associated SARI cases in province Y (including base province)

$P{op}_{Y}$= Population in province Y (including base province)
